# COVID-19 vaccine-induced parkinsonism due to LGI1 antibody encephalitis: case report and brief literature review

**DOI:** 10.1093/omcr/omaf236

**Published:** 2025-11-26

**Authors:** Abdalla Khabazeh, Jetish Kumar, Volney Sheen

**Affiliations:** Faculty of Medicine, Damascus University, 3 Almazzeh Rd, Damascus, Syria; Department of Neurology, Beth Israel Deaconess Medical Center and Harvard Medical School, 330 Brookline Avenue, Boston, MA 02115; Department of Neurology, Beth Israel Deaconess Medical Center and Harvard Medical School, 330 Brookline Avenue, Boston, MA 02115

**Keywords:** autoimmune, COVID-19, encephalitis, parkinsonism, tremor, vaccination

## Abstract

Anti-LGI1 encephalitis is an autoimmune disorder of the brain, characterized by subacute cognitive impairment, faciobrachial dystonic seizures, and hyponatremia. Although rare, recent reports suggest that LGI1 encephalitis may be triggered following COVID-19 exposure whether through infection or vaccination. It usually presents with insidious progression which, along with old age predominance, may delay the diagnosis. We herein report a 67-year-old patient with positive LGI1 antibody titers, who developed subacute parkinsonism after serial COVID-19 vaccination. To our knowledge, this is the first documented case report highlighting a potential association between COVID-19 vaccination and the development of parkinsonism in the context of LGI1 encephalitis.

## Introduction

Anti-LGI1 (leucine-rich glioma-inactivated protein 1) antibody-associated encephalitis is a rare autoimmune disorder affecting the brain but is considered the most common cause of autoimmune encephalitis in adults older than 40 years with male predominance [1]. It typically presents with cognitive impairment, faciobrachial dystonic seizures (FBDS), as well as possible refractory hyponatremia [2, 3]. In very rare instances, it has been associated with the development of neurodegenerative disorders, such as Parkinson’s disease. However, none of the previous studies have reported parkinsonism development following COVID-19 vaccination in the context of LGI1 encephalitis. Although its pathophysiology remains incompletely understood, it is considered a subtype of limbic encephalitis usually occurring without any detectable paraneoplastic cause [4].

We herein report the case of a 67-year-old man who developed subacute parkinsonism following serial COVID-19 vaccination, with anti-LGI1 encephalitis emerging as a potential mediating factor. This case report aims to contribute to the growing literature on LGI1 encephalitis following COVID-10 vaccination, emphasizing the possibility of the development of parkinsonism features in the context of this disorder.

## Case report

A 67-year-old male presented with a six-month history of involuntary left-hand tremors, described as rhythmic, worsening at rest, and improving with movement. There is no personal or family history of tremors, Parkinson’s disease, or any other neurological conditions.

**Figure 1:**
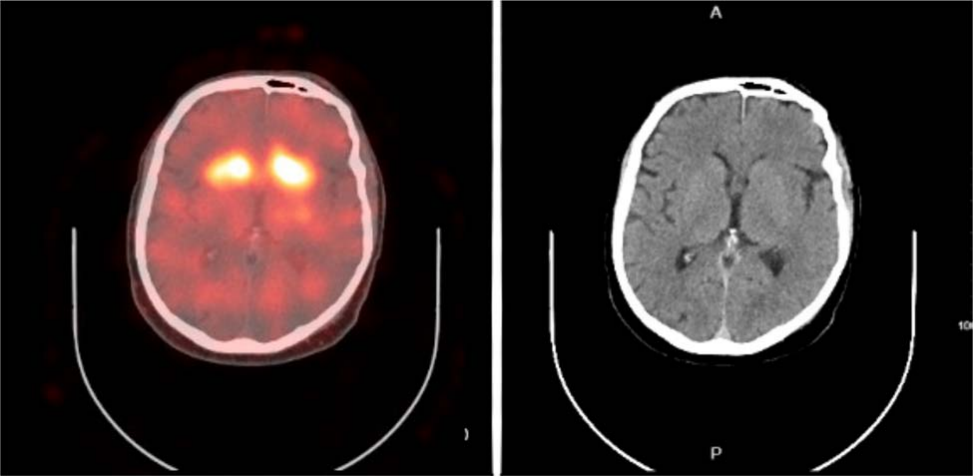
SPECT/CT images show decreased tracer activity in the bilateral putamina.

The patient received the Moderna COVID-19 vaccine and a subsequent booster early this year. About one week after the first dose, he developed a left-hand and mouth tremor that progressively worsened following the booster, accompanied by occasional mouth spasms. Examination revealed mild hypomimia, a left-sided resting tremor, bradykinesia, and rigidity, but no hypophonia or postural instability.

Laboratory tests were normal except for mild hyponatremia (Na: 132 mEq/L). CSF analysis was recommended but declined by the patient. MRI was contraindicated due to retained shrapnel from a prior traumatic brain injury; therefore, SPECT/CT brain imaging was performed, demonstrating decreased tracer activity in the bilateral putamen (Fig. 1), consistent with parkinsonism. Workup began when the patient presented for his annual seizure follow-up and reported persistent tremor. Given the possible association with COVID-19 vaccination, an autoimmune encephalopathy panel was sent, confirming positive LGI1 antibodies and leading to a diagnosis of LGI1 encephalitis with parkinsonism. The patient was offered Sinemet for motor symptoms, as he declined steroid therapy. Sodium levels were monitored, and treatment was adjusted based on clinical response. Physical therapy was initiated to support motor function and mobility.

## Discussion

Anti-LGI1 encephalitis has classically been associated with cognitive changes, faciobrachial spasms/seizures and hyponatremia which is usually present in 60–70% of patients due to autoantibody-binding to LGI1-expressing, ADH-secreting neurons [5]. Parkinsonism can be seen in up to 11% of patients with this condition, which may be explained by autoimmune-mediated disruption of the basal ganglia, leading to dopaminergic dysfunction and motor symptoms resembling parkinsonism.

While pathophysiology remains unclear, it shares similarities with post COVID-19 infection encephalitides, suggesting a common mechanism. Antibodies to NMDAR, GAD, and MOG have been linked to both infection and vaccination, while CASPR-2 and IgLON5 are only associated with infection. CASPR-2 and LGI1 are key components of the Kv1 potassium channel complex [6]. Genetic predisposition also plays a role, as anti-LGI1 encephalitis is linked to the DRB1*07:01-DQB1*02:02 haplotype in HLA class II and B*44:03 in HLA class I [7]. This suggests complex interplay between environmental exposure from COVID-19 infection/vaccination and the innate immune system.

LGI1 encephalitis was well described in medical literature. One retrospective study (2013–2021) from South India included 23 patients with LGI1 antibodies who showed progressive slowness, gait disturbances, and paroxysmal episodes of falls resembling Parkinsonism [8]. Another study reported two patients developing severe dyskinesia after the BNT162b2 COVID vaccine, with one experiencing fever, confusion, and persistent dyskinesia for three days [9]. In contrast, our patient had mild Parkinsonism-isolated tremors which progressed insidiously, delaying the diagnosis.

Management is based on immunotherapy, with corticosteroids and/or IVIG as first-line options. Dopaminergic agents like levodopa are used for parkinsonism, though clinical responses vary. A multidisciplinary approach, including neurologic follow-up and rehabilitation, is essential for optimizing recovery and long-term outcomes.

## Conclusion

This case highlights that, COVID-19 vaccination can potentially trigger autoimmune LGI1 encephalitis and subsequent parkinsonism. In patients with recent COVID-19 exposure, whether through infection or vaccination, awareness of the development of parkinsonism features should prompt clinicians to consider the possibility of an underlying autoimmune encephalitis and guide diagnostic procedures.
